# Recent Insights Into the Role of Macrophages in Acute Gout

**DOI:** 10.3389/fimmu.2022.955806

**Published:** 2022-07-08

**Authors:** Lei Liu, Lingjiang Zhu, Mengdan Liu, Li Zhao, Yiyun Yu, Yu Xue, Lizhen Shan

**Affiliations:** ^1^ Department of Rheumatology, The Second affiliated Hospital of Zhejiang University, School of Medicine, Hangzhou, China; ^2^ Division of Rheumatology, Huashan Hospital, Fudan University, Shanghai, China; ^3^ Department of Endocrinology, The Second affiliated Hospital of Zhejiang University, School of Medicine, Hangzhou, China

**Keywords:** synovial resident macrophage, lining layer, gout, spontaneous resolution, monocyte derived macrophage

## Abstract

Gout is a common type of inflammatory arthritis characterized by the presence of monosodium urate crystals (MSU) in the joints. Macrophages are believed to be involved in gout flares. It has long been recognized that resident macrophage and monocyte derived macrophages are distinct subsets and there have been attempts to investigate their roles in acute gout, respectively. Previous studies revealed that resident macrophages initiate and drive the inflammation, while monocyte derived macrophages differentiated into M1-like macrophages in response to MSU crystals. With the advancement of technologies, subpopulations of synovial resident macrophages have been defined with the characteristics more accurately described. Resident macrophages in the synovial lining layer showed an anti-inflammatory effect in rheumatoid arthritis, but specific *Trpv4* depletion of them reduced MSU crystals induced murine arthritis. CD14^+^ monocytes in the synovial fluid from patients with gout exhibit phenotypes of anti-inflammatory as well as pro-inflammatory characteristics. Here, we review the main aspects of macrophages in the initiation and resolution of acute gout and try to clarify the specific role of each subpopulation. Building a reliable diagram of the effect of monocytes and macrophages during MSU crystals induced arthritis will bring us closer to targeting macrophages for improving the management of gout.

## Introduction

Acute gout is an auto-inflammatory disease characterized by self-limiting inflammation in response to the deposition of monosodium urate (MSU) crystals in the joints (1). Various immune cells are involved in the initiation, development and spontaneous resolution. The role of macrophages, neutrophils (2–5), mast cells (6, 7) and, less frequently mentioned previously, T cells (8–10) have been revealed by recent studies. The macrophage is one of the earliest recognized and well-studied immune cells that mediated the entire process of acute gout. However, there are several issues that have been debated. The ultimate one is the role of resident macrophages and monocyte-derived macrophages in acute gout. In the meantime, as the study of synovial macrophages has been advanced, methods like fate-mapping approaches and single-cell RNA sequencing (scRNAseq) have revealed more refined and distinct phenotypes and functions of the synovial resident macrophages (11–13). It has allowed a great progress in the study of rheumatoid arthritis (RA). However, in the field of acute gout, there is still no consensus, and this review attempts to elucidate the role of these two subgroups of macrophages in the process of acute gout by combining the recent studies.

## The synovial resident macrophages are divided into three subpopulations

The synovium is a thin membrane encapsulating the joints and supports its mobility. It consists of two layers, the intimal lining layer composed of fibroblast-like synoviocytes and macrophage-like synoviocytes. And the sub lining layer is the underlying connective tissue composed of fibroblasts, fat cells, macrophages and blood vessels (14). Synovial tissue resident macrophages were previously identified by CD68^high^CD163^high^ (15). Lining synovial macrophages with the capacity of cellular clearance were discriminated from sub-lining cells by the expression of FcγRIIIa (16, 17). However, contradictory findings (18–20) call for more adequate and precise markers to discriminate between the various subgroups of synovial resident macrophages.

A recent approach using fate mapping and reporter mice in conjunction with three-dimensional light sheet microscopy yielded important insights into the spatiotemporal composition, origin and biology of synovial resident macrophage subsets (13). Lining macrophages (the major resident group) selectively express CX3Cr1 and organize in dense membrane-like barrier structures. They also show an immune regulatory and highly phagocytic phenotype with the expression VSIG4 (21) and TREM2 (22). MHCII^+^ interstitial macrophages are in the sub lining layer and give rise to the CX3Cr1^+^ macrophages. A third population of RELMα^+^CD163^+^CD206^+^ interstitial macrophages, also generated from the MHCII^+^ interstitial macrophages, display many features of alternatively activated macrophages. Additional studies have subsequently demonstrated similar synovial macrophage phenotypes, from both human and murine perspectives (11, 12).

## Synovial resident macrophages mainly initiate and drive MSU crystals induced inflammation

Wiliiam John Martin and colleagues confirmed resident macrophages initiating and driving inflammation in MSU crystals induced murine peritoneal models (23). This study served as a cornerstone for the role of resident macrophage in gout. For more than a decade thereafter, studies on both the mechanisms of acute gout and intervention strategies have been conducted on the basis of the latter research. However, the conclusion was sometimes challenged. From a macrophage classification perspective, the peritoneal cavity could somehow mimic the joint, but peritoneal macrophages are not equivalent to synovial macrophages. An investigation of synovial fluid mononuclear cells in patients with acute gout revealed that CD14^+^ infiltrated monocytes rather than tissue-resident macrophages produced pro-inflammatory cytokines (24). Although a sparse number of macrophages phenotypically representative of resident macrophages were present in the synovial fluid, they do not truly characterize the function of synovial resident macrophages, either.

What really makes the theory that resident macrophages initiate and drive MSU crystal-induced arthritis challenged is that multiple studies have indicated the synovial lining macrophages display an immune regulatory phenotype and effect. Depleting of the synovial CX3CR1^+^TREM2^+^ macrophages aggravated the arthritis in K/BxN STA murine models (13). Two synovial macrophage subpopulations (MerTK^pos^TREM2^high^ and MerTK^pos^LYVE1^pos^) from patients with rheumatoid arthritis (RA) showed unique remission transcriptomic signatures enriched in negative regulators of inflammation (11). HUANG and colleagues observed that the synovial macrophage tissue–resident niche is necessary for suppression of chronic inflammation and may contribute to the pathogenesis of RA (12). All these studies were conducted to investigate the contribution of synovial lining macrophages in arthritis and invariably identified their anti-inflammatory and tissue stabilizing properties. The latter evidence leads us to wonder whether synovial resident macrophages actually initiate and drive acute gout, and whether the numerous studies on them as target cells were rational.

Notably, the above studies all described the role of synovial resident macrophages in chronic arthritis like RA. In contrast, gout is a crystal-induced acute arthritis. An important investigation by Zhou Lan et al. confirmed that TRPV4 is critically involved in the activation of macrophage NLRP3 inflammasome and production of interleukin(IL)-1β induced by MSU crystals (25). In the study, they established the macrophage-specific *Trpv4 cKO* (*Cx3cr1^CreERT^; Trpv4^f/f^* ^)^ murine model. Fate mapping studies have shown that tissue-resident macrophages retained their identity but the monocyte-derived macrophages were replaced with wild type macrophages produced by bone-marrow-derived progenitors in the inducible macrophage-specific *Trpv4 cKOs* four weeks after tamoxifen administration (25). The ankle swelling was significantly reduced in the *Cre^+^Cx3cr1^CreERT^*; *Trpv4^f/f^* mice when compared with their Cre^-^ littermates. TPRV4 has been known to aggravate inflammation by activating NLPR3 inflammasome. And TPRV4 could not work if the CX3CR1^+^ synovial macrophages did not undergo a pro-inflammatory response with MSU crystals. Therefore this study provided sufficient evidence for people to believe that CX3CR1^+^ synovial resident macrophages initiate and drive MSU crystal-induced arthritis.

There has been no direct evidence to prove the involvement of synovial resident macrophage in the spontaneous resolution of acute gout. In our previous study (26), we observed that in rat gouty joints, the M2 (alternative macrophage polarization) was seen at an early stage at 2 hours after injection of MSU crystals. And in the murine peritoneal model, the anti-inflammatory cytokine IL-37 level also peaked at 2 hours after injection of MSU crystals. RELMα^+^CD163^+^CD206^+^ interstitial macrophages express genes associated with alternative activation, suggesting an immunosuppressive phenotype that is protective. We suppose they might be the source of M2 at an early stage of acute gout and involved in the initiation of the spontaneous resolution. However, the exact positioning of synovial RELMα^+^ interstitial macrophages requires further investigation by imaging and functional technologies.

## Recruited monocytes may exhibit both pro-inflammatory and anti-inflammatory phenotypes in MSU induced arthritis

Macrophages were originally proposed to be derived from circulating monocytes (27). With the advancement of research tools, such as lineage tracing (28, 29), depleting strategies (30, 31), therapeutic targeting (28) and monitoring (32), it has been accepted that many tissue macrophage populations arise during embryonic development, are seeded well before birth and are thereafter maintained by local proliferation rather than monocyte recruitment (33). Circulating monocytes are completely or at least partly independent from resident macrophages. Consequently, when we consider the tissue inflammation, it is important to investigate the precise effect of resident macrophages and monocyte-derived macrophages, respectively. The classical hierarchical mononuclear phagocyte system proposed by Van Furth et al. in 1972 held the opinion that circulating monocytes from the bone marrow continuously migrated to peripheral tissues, where they differentiated into macrophages and then worked (34). However, many researchers discovered that the monocytes were also involved in the inflammatory responses (35–39).

Several studies have shown the phenotype of the circulating monocytes during MSU induced inflammatory responses (40–43). Only a few directly explored the role of monocytes in the joints during acute gouty inflammation. We have to recognize that cells with an intermediate immunophenotype are detected during the differentiation and maturation from monocytes to mature macrophages. The phenotype of monocyte-derived macrophages is affected by the phase of inflammation that affects the composition of macropahges with different maturation stages. Therefore, we need to pay great attention to the time point of the studies on monocyte phenotypes when discussing these researches.

By using the murine peritoneal model, William John Martin proved MSU crystal-recruited monocytes differentiate into pro-inflammatory M1-like macrophages *in vivo (* 44). In this study, the most straightforward evidence was that the expression of pro-IL-1β, IL-1β, pro-caspase-1 and caspase-1 of the cells from the peritoneal fluid increased with time. However, what has been troubling us is that the expression of the above proteins was highest at 72 hours after MSU crystal injection, which meant a very late phase of acute gout. But at 18 hours, the inflammation in the peritoneal cavity had all resolved and was no longer occurring. How did these cells with high expression of inflammasome related proteins play a non-inflammatory role? Are these really monocyte-derived macrophages or just the resident macrophages which were previously attached to the peritoneum returning to the peritoneal cavity?

In patients with gout, CD14^+^ monocytes were markedly increased in the synovial fluid (24). The CD14^+^ monocytes showed the phenotypes of infiltrated monocytes rather than tissue-resident macrophages. Intracellular cytokine staining of the CD14^+^ monocytes showed considerable amounts of IL-8. However, there was no significant increase of pro-inflammatory cytokines after MSU re-stimulation. Compared with monocytes from patients with RA, monocytes of gouty patients produced more IL-10 significantly. Thus, the authors came to a conclusion that CD14^+^ cells of the synovial fluid from the patients with gout are infiltrated monocytes and exhibit phenotypes of anti-inflammatory as well as pro-inflammatory characteristics. Since the samples of joint fluid were obtained from patients with gout, the authors only stated that the patients were in active disease at the time of sampling, and there was no specific time point, so there was variability in the phenotype ofmonocytes from various patients. In our own study, we placed great emphasis on the phenotypic changes of monocyte-macrophages with time changes following MSU crystal intervention, we have also discovered that the iNOS/Arg-1, which represented M1/M2 ratio, of peritoneal monocytes increased with time post MSU crystals injection (26). It means that M1 and M2 co-existed in the inflamed tissue and the number of M1 exceeded M2 at the initial and progressing stages of acute gout.

## Discussion

It is believed that macrophages initiate and drive the MSU induced arthritis. And a variety of new therapeutic targets focusing on macrophages have been constantly discovered. Our group has published articles describing the role and mechanism of Sirt1, IL-37 and leptin on macrophages in mediating acute gout (45–49). Also, we observed distinct macrophage polarizations in acute and chronic gout (26). It is with the accumulation and continuous progress of studies that we believe it is necessary to properly characterize the role of macrophages, especially the various subpopulations, in acute gout and the spontaneous resolution.

**Figure 1:**
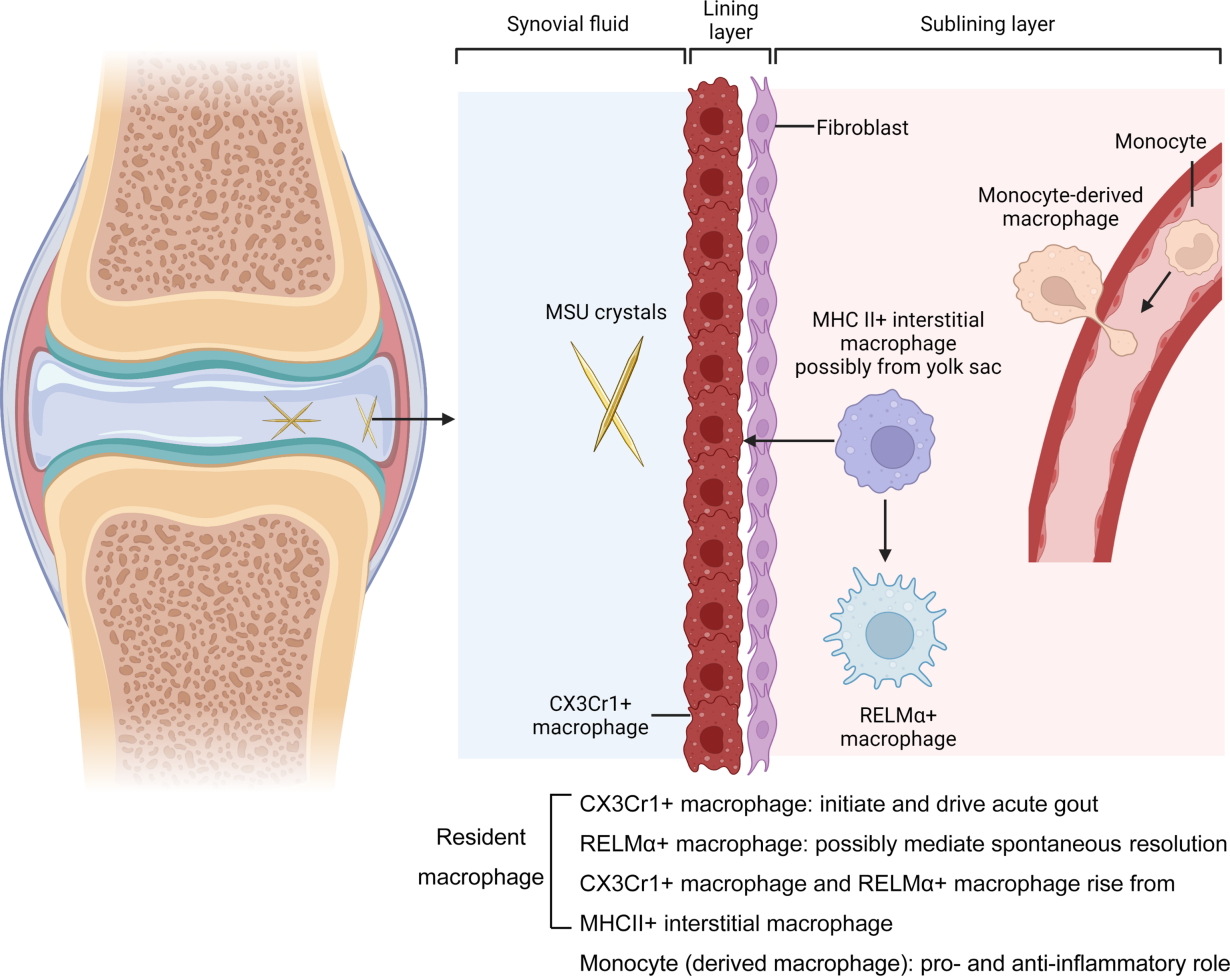
Macrophages in the MSU crytals induced arthritis. Joints are surrounded by a tissue called the synovium, which is formed from layers of cells called the lining and the sublining layers. The resident macrophages in the lining layer highly expressed CX3Cr1 and form a barrier adjacent to a layer of cells called fibroblasts. They initiate and drive acute gout. Barrier-forming macrophages arise from a subpopulation of MHCII^+^ macrophages called interstitial macrophages in the sublining layer. The MHCII^+^ macrophages aslo generate another subset called RELMα^+^ interstitial macrophages. They express genes associated with alternative activation (CD163^+^CD206^+^), suggesting an immunosuppressive phenotype that is protective. The other Non-resident macrophages enter the joint from blood vessels. These cells, which can drive and supress MSU crystals induced inflammation, derived from monocytes. Mr. Xiangyu Xu and Dr. Lei Liu, created with BioRender.com.

When we searched “gout and macrophage” on the Pubmed from 2007 to 2022, there were 336 articles. Only a few of them actually elucidate the role of resident macrophages, monocytes and monocyte-derived macrophages in acute gout. The fate-mapping approaches and scRNAseq have divided the synovial resident macrophages into three well-defined subpopulations, of which the major one is the lining CX3Cr1^+^ macrophages. The other two sub-populations are the MHCII^+^ interstitial and RELMα^+^CD163^+^CD206^+^ resident synovial macrophages in the sub-lining layer, see **Figure 1**. While studies have implicated the lining CX3Cr1^+^ macrophages in maintaining joint homeostasis and suppressing chronic arthritis, a recent study confirmed that they do initiate MSU crystals induced arthritis (25). Taking into account our own findings, we propose that RELMα^+^ CD163^+^CD206^+^ resident synovial macrophages may directly generate M2 macrophage polarization and mediate the spontaneous resolution, “ programmed” for remission at the onset of MSU crystal induced inflammatory responses. Although sparsely studied and in contrast to the role played in chronic arthritis, the available evidences all suggests that synovial resident macrophages initiate and drive the MSU crystal induced inflammatory responses, with a minor proportion, probably RELMα^+^CD163^+^CD206^+^ interstitial macrophages, mediating the resolution of inflammation at an early stage.

Studies on monocytes in acute gouty arthritis are even fewer and there seemed no consensus among the researchers on this issue. The most widely accepted finding comes from the MSU crystal-induced murine model of peritoneal inflammation (44). Although there were results suggesting that the recruited monocyte-macrophages expressed higher levels of both IL-1β and caspase-1 over time. The major problem was that the phenotype of the cells was not consistent with the overall phenotype of the murine model, and the inflammation in the peritoneal cavity had spontaneously resolved at the time when monocyte-macrophage expression of pro-inflammatory factors was at the highest level. We suppose that this part of the study may not be truly representative of the features of the recruited monocytes. A recent study explored the phenotype and function of CD14^+^ monocytes in the synovial fluid of patients with acute gout (24). What is indicative of the pro-inflammatory profile of the recruited monocytes is the elevated intracellular IL-8 level. But they did not secrete more pro-inflammatory cytokines than the control group when re-stimulated by MSU crystals *in vitro*. Interestingly, this group of cells also expressed the surface markers of M2, suggesting that the recruited monocytes might also play a role in suppressing inflammation in the joint. We have also observed M2 from the peritoneal fluid at the early stage of MSU crystals induced peritonitis (26).

In addition to the fact that direct observation of various subpopulations of macrophages in the joint is the most crucial challenge to clarify the topic, the lack of consensus on origin of macrophages and how to derive and activate them with MSU crystals is another important issue. THP-1 designates a spontaneously immortalized monocyte-like cell line, which has been widely used in studies of gout. Pam3CSK4 (50), PMA (51), LPS (52), and IL-6 (53) were all used for priming the THP-1 cells. Bone marrow derived monocytes (BMDM) are murine primary cells, for which priming is necessary as well. The colony-stimulating factors, macrophage colony-stimulating factor (M-CSF) and granulocyte macrophage-colony stimulating factor (GM-CSF), are known to prime or activate BMDM (54). However, they have substantial polarizing effects (55). Therefore, distinct protocols bring different effects on macrophages derived from primary monocytes or cell lines. This makes the macrophage status prior to the MSU crystal intervention vary and is poorly comparable across various studies. An important nomenclature and experimental guideline for macrophage activation and polarization was raised by Peter J. Murray and colleagues (56). In the fields of studies of gout, we hope standardization issues for priming and activating the monocytes could be established and accepted to avoid stunting progress. Importantly, in our studies, non-primed murine peritoneal resident macrophages and RAW264.7 cell lines exhibited a pro-inflammatory phenotype in response to MSU crystals (45, 47, 48). It has been proved that MSU crystals alone induce a metabolic-inflammatory transcriptional program in non-primed human and murine macrophages that is markedly distinct to that induced by LPS. Increased binding of JUN to the promoter of target genes through JNK signaling initiates the inflammatory-metabolic changes (57). Nonetheless, some studies still primed the macrophages and resulted in more robust inflammatory responses. It is also important to note that the inflammatory response of macrophages to MSU crystals is not equal to that of LPS and special regard should be paid when designing studies. Therefore, the consensus of primary monocytes and macrophages and the corresponding cell lines appear to be crucial for the future study of gout.

Although there are numerous studies on the anti-inflammatory treatment of gout, the only agents currently available for clinical use are colchicine, NSAIDs, glucocorticoids, and anakinra, of which, the side effects constrain their use in patients with cardiovascular, gastrointestinal, or renal comorbidities. Clinical translational medicine is inseparable from advances in disease mechanism research. Currently in the field of acute gout, the role of diverse subpopulations of macrophages, one of the most important targets of gout therapy, in the various stages of initiating, driving and spontaneous resolution have not well been understood. With the development of techniques, such as fate-mapping, scRNAseq and three-dimensional light-sheet fluorescence microscopy, we believe that people will eventually have a clear understanding of the relationship between macrophages and gout and obtain better ideas for the treatment of the disease.

## Author Contributions

LL, YY, LJZ, and LZ drafted the manuscript. LS and YX supervised and edited the manuscript. All authors contributed to the article and approved the submitted version.

## Funding

This work was supported by the National Natural Science Foundation of China No. 81801597.

## Conflict of Interest

The authors declare that the research was conducted in the absence of any commercial or financial relationships that could be construed as a potential conflict of interest.

## Publisher’s Note

All claims expressed in this article are solely those of the authors and do not necessarily represent those of their affiliated organizations, or those of the publisher, the editors and the reviewers. Any product that may be evaluated in this article, or claim that may be made by its manufacturer, is not guaranteed or endorsed by the publisher.
